# Smartphone-based multi-criteria vegetable object detection dataset from Bangladesh

**DOI:** 10.1016/j.dib.2025.112281

**Published:** 2025-11-14

**Authors:** Sabrina Jahan, B.M. Shahria Alam, Ishraque Manzur, Tawhidur Rahman, Mahamudul Hasan, Raiyan Gani, Md Miskat Hossain, Karib Shams, Mohammad Rifat Ahmmad Rashid

**Affiliations:** Department of Computer Science and Engineering, East West University, Aftabnagar, Dhaka, Bangladesh

**Keywords:** Vegetable detection, Imaging dataset, Vegetable identification, Computer Vision, Agricultural Practices

## Abstract

The agricultural landscape of Bangladesh is significantly influenced by the cultivation of vegetables, which is directly involved in the nutritional intake of the people, the economy, and the food security of the entire nation. Precise identification of vegetables is essential to efficient cultivation, inventory management, and smart agricultural practices. In our study, we introduce a comprehensive dataset for vegetable detection, consisting of 3534 high-resolution images captured in natural, real-world settings using a Redmi Note 12 from multiple roadside vehicles of local vendors. The dataset encompasses 22 distinct vegetable classes, covering a wide range of appearances, shapes, and natural daylight environments to enhance model robustness and practical applicability. Each image has been meticulously annotated using the Roboflow platform to facilitate object detection tasks, and the resulting dataset is provided in Pascal VOC format. Unlike other imaging datasets, our work emphasizes ground-level perspectives, making it particularly relevant for handheld and low-cost monitoring systems. The primary goal of this dataset is to support the development of computer vision models for accurate vegetable recognition, thereby aiding decision-making in vegetable cultivation and contributing to smarter and sustainable agricultural practices.

Specifications Table

**Table:** 

Subject	Plant Pathology is a branch of Agriculture
Specific subject area	Automated vegetable detection leveraging computer vision techniques
Type of data	Images and XML file
Data collection	We gathered data from Banasree, Block# C, Road no 07, Rampura, Dhaka, using a smartphone equipped with a 50 MP rear camera. The device features a Qualcomm Snapdragon 685 (6 nm) chipset and captures video with high clarity under natural daylight. Footage was recorded and later processed by extracting high-resolution frames. In order to achieve accurate and reliable annotations, we used the Roboflow platform [13] for our work. The original dataset consists of 3534 images. Blurred or obstructed visuals were excluded to ensure dataset quality and consistency.
Data source location	Institution: East West University City/Town/Region: Banasree, Block #C, Road No 07, Rampura, Dhaka Country: Bangladesh Latitude and Longitude: Banasree, C block: Latitude 23.7619° N, Longitude 90.4331°
Data accessibility	Repository name: Mendeley Data Data identification number: 10.17632/gnc4s3z2mf.3 Direct URL to data: https://data.mendeley.com/datasets/gnc4s3z2mf/3 Instructions for accessing these data: Visit the Mendeley Data portal. • Click the “Download All Files” button. • Once downloaded, unzip the archive. You will find the Original Data folder containing the train, test, and valid subfolders. • Use these subfolders directly in your machine learning or deep learning pipelines as required.
Related research	None

## 1 Value of the Data

• Provides a benchmark dataset of 22 vegetable classes collected in real-world conditions, enabling reliable model training and evaluation for agricultural computer vision tasks.
• Supports automation in farming and food processing, such as harvesting, freshness detection, ripeness evaluation, and foreign-object detection, reducing reliance on manual labor.
• Facilitates precision agriculture in Bangladesh and beyond by enabling cost-effective, data-driven yield management and quality control.
• Ensures compatibility and reproducibility by using standard annotation formats (Pascal VOC, COCO), allowing comparative studies across frameworks.
• Offers a resource for lightweight and scalable deployment of models in resource-constrained environments (e.g., farms, handheld devices, retail).
• Strengthens food safety and supply chain management by enabling detection of contaminants and improving product quality evaluation.
• Provides a foundation for future research, including development of novel detection models, ensemble architectures, and semi-supervised learning strategies.

## 2 Background

The rapid integration of computer vision and deep learning into agriculture has driven substantial advances in fruit and vegetable detection, classification, and quality assessment. Prior works highlight the potential and challenges of deploying robust models in diverse agricultural environments. For instance, the FRUVEG67 dataset introduced 67 classes of fruits and vegetables, accompanied by a semi-supervised annotation method and the ensemble YOLOv7-based FVDNet, which achieved an mAP of 0.78 across all classes, advancing object detection in unconstrained conditions [1]. Similarly, YOLO-Vegetable, an improved YOLOv10 variant, addressed greenhouse-specific challenges such as uneven illumination and target occlusion, showcasing how architectural innovations enhance agricultural detection accuracy [2].

Beyond classification, real-time object detection has been applied to automated harvesting systems, where deep learning models improve both speed and accuracy of vegetable identification, reducing manual labor dependency and improving scalability in large-scale farming operations [3]. A dataset for vegetable identification was introduced, containing nearly 4800 images of six vegetable species captured under diverse environmental settings. The dataset supports research in vegetable recognition and detection tasks, especially in unconstrained conditions with varying backgrounds and lighting, making it a valuable benchmark for computer vision in agriculture [4].

The adoption of standard annotation formats (Pascal VOC, COCO) plays a vital role in dataset interoperability and reproducibility, allowing comparative benchmarking across multiple frameworks [5]. The EA-CNN framework, an enhanced Attention-based Convolutional Neural Network with Explainable AI integration, was proposed for fruit and vegetable recognition. By combining enhanced attention mechanisms with customized pooling, the model captures fine-grained visual features. EA-CNN was tested on the Fruit-360 and Fruit Recognition datasets, and achieved 98.1 % and 96 % accuracy respectively, outperforming conventional CNNs and attention-based models. Moreover, the integration of explainable AI techniques improves interpretability, allowing clearer insights into the model’s decision process [6].

The Potato Detection Dataset (v11, 2023) was developed to support vegetable detection tasks in agricultural settings. It includes 8034 annotated images of potatoes captured in real-world environments, enabling the training and evaluation of object detection models. The images were pre-processed to 640 × 640 resolution and enhanced through augmentations such as flips, rotations, blur, and noise, ensuring robustness against diverse conditions [7]. Lightweight frameworks have been introduced to simplify recognition tasks, enabling model deployment in resource-constrained agricultural environments [8]. Furthermore, integrating image processing with deep learning has enhanced automated quality assessment pipelines, improving efficiency and consistency in production systems [9]. Other architectures, such as FVRT-DETR, which detects 40 classes of fruits and vegetables, and hybrid deep learning models combining VGG-16, VGG-19, and Inception V3, further highlight the scalability and performance potential of detection and classification in agriculture [10,11]. Together, these contributions underscore the value of curated datasets and advanced models in enabling automation, safety, and efficiency in agriculture.

The present dataset builds on these developments by focusing on 22 vegetable classes relevant to Bangladesh, captured under real-world farming conditions with smartphone-based imaging. The dataset is thoroughly annotated using Roboflow, ensuring precise labeling for training modern deep learning models. This resource is intended to support empirical research in vegetable detection, quality control, and cost-effective yield optimization, aligning with global efforts to strengthen digital and data-driven farming.

## 4 Data Description

The dataset is assembled from a total of 3534 images of annotated vegetables. It consists of 22 classes, namely Beetroot, Bitter Gourd, Bottle Gourd, Cabbage, Capsicum, Carrots, Cauliflower, Coriander leaves, Cucumber, Eggplant, Green Banana, Green Beans, Green Chilli, Green Papaya, Lemon, Potato, Pumpkin, Radish, Snake Gourd, Spring Onion, Tomato and Turnip. All the annotations, splitting, pre-processing were done using Roboflow [12], which is a web-based data annotation tool. In the dataset, each category represents a distinct class, encompassing a wide variety of instances. The frequency of instances per category varies which reflects natural diversity in real world datasets. Table 1 provides a summary of the number of object instances for each class, highlighting the distribution and relative representation of categories within the dataset.

**Table 1:** Distribution of annotated object instances across 22 vegetable classes in the dataset.

Class Name	Instances Count
Beetroot	1747
Bitter Gourd	3164
Bottle gourd	2689
Cabbage	1678
Capsicum	2581
Carrots	2908
Cauliflower	3098
Coriander leaves	4113
Cucumber	12,873
Eggplant	6274
Green Banana	583
Green Beans	7468
Green Chilli	5329
Green Papaya	3072
Lemon	6247
Potato	2994
Pumpkin	3720
Radish	791
Snake gourd	1227
Spring Onion	1509
Tomato	11,737
Turnip	4352

**Fig. 1:**
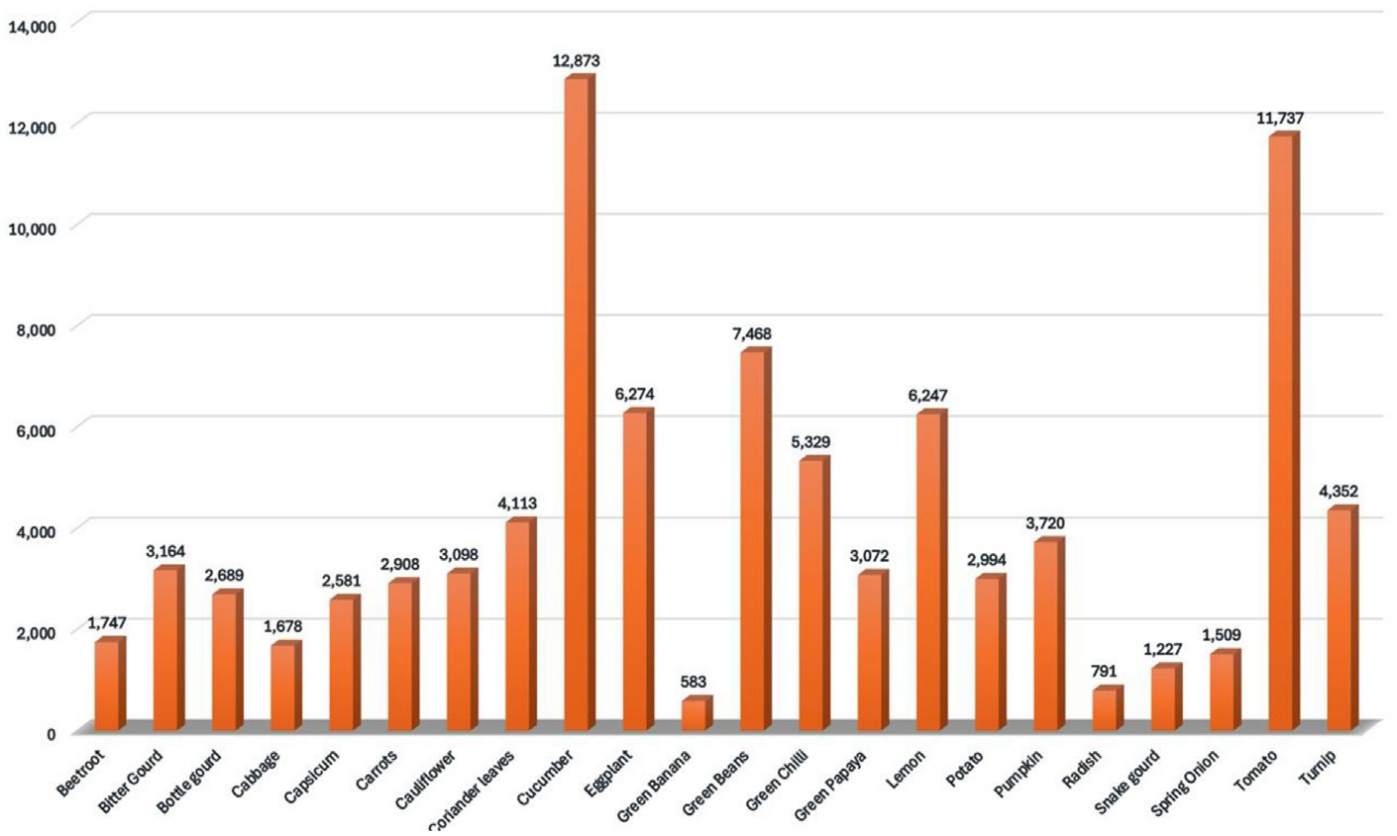
Distribution of vegetable instances across different categories.

As shown in Fig. 1, the dataset contains a varying distribution of vegetables across multiple categories.

**Fig. 2:**
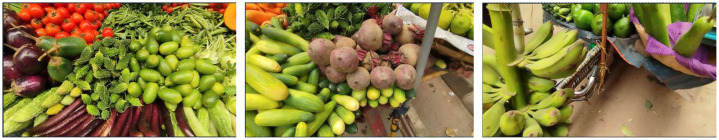
Representative images from various vegetable classes.

For more accurate detection [11], the images were taken from different angles and in natural lighting with real-world environment and the images contain bounding boxes for each kind of vegetable. Fig. 2 shows some samples of images from some classes.

**Fig. 3:**
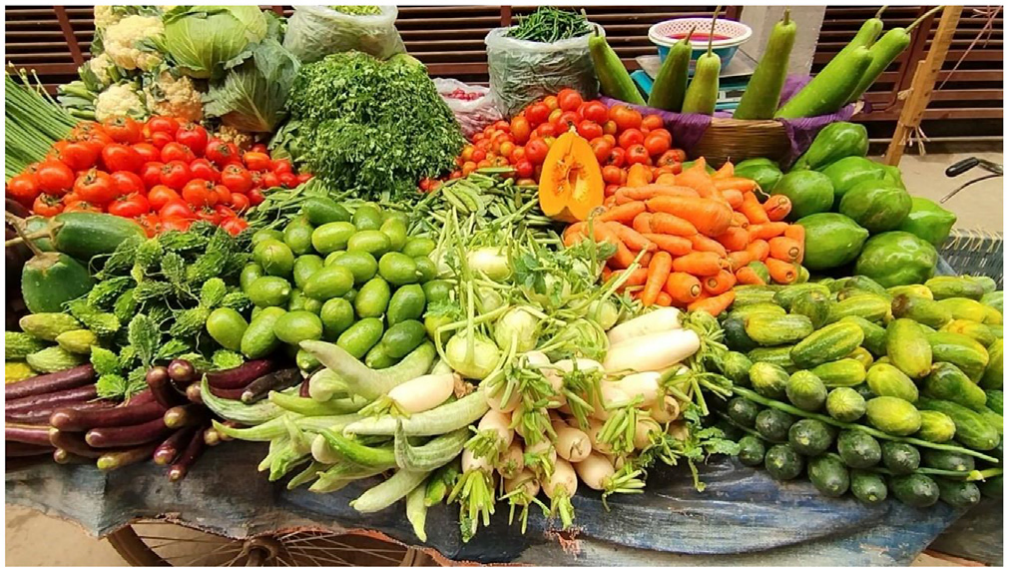
Sample Vegetable Image.

**Fig. 4:**
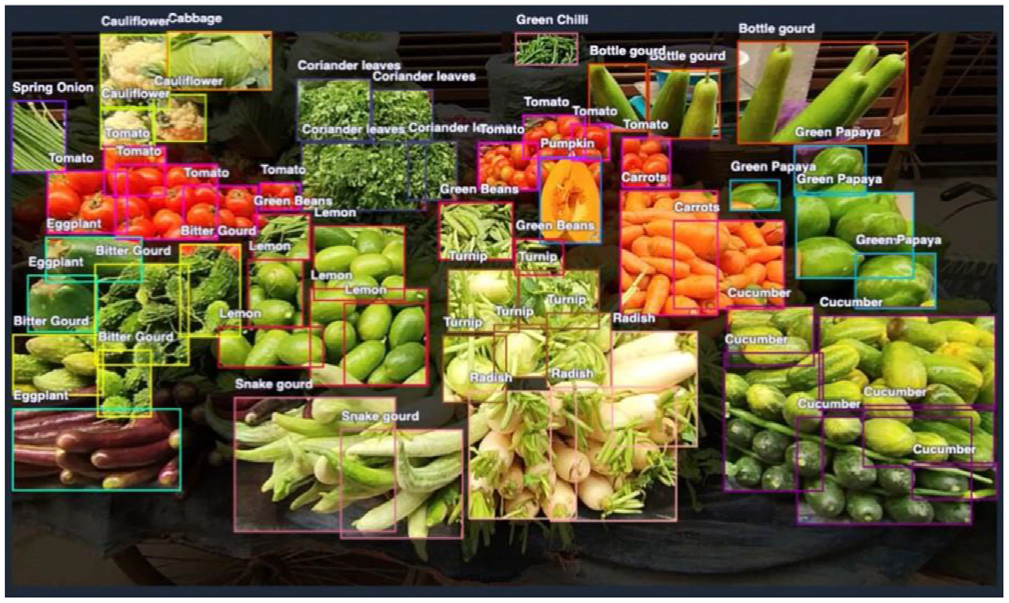
Sample Vegetable Image with Annotations.

The images in the dataset are set in JPG format, and all the annotations are also provided in Pascal VOC XML format. In the Pascal VOC format, each annotation [5] defines object locations using coordinates of the top-left corner along with the corresponding width and height, providing a standardized structure for representing bounding boxes. The images were initially captured in MP4 video format before being processed to be annotated [4] in Roboflow. Each annotation file contains the image ID and associated label names for precise categorization of vegetables. Fig. 3, Fig. 4 demonstrate a sample image of the vegetables in both unannotated and annotated forms, highlighting the precision and usefulness of the applied annotation techniques.

The initial dataset was collected [2] using a single Android smartphone, and later on, the images were processed for further processing for annotation. After annotating the images in the dataset, the initial pre-processing was simple, as it was applied to enhance the quality of the dataset. Pre-processing, such as resizing, was used for practicality. This makes sure the dataset is diverse, as it contains multiple classes of vegetables, making it more realistic for real-world practicability and model training [9].

**Fig. 5:**
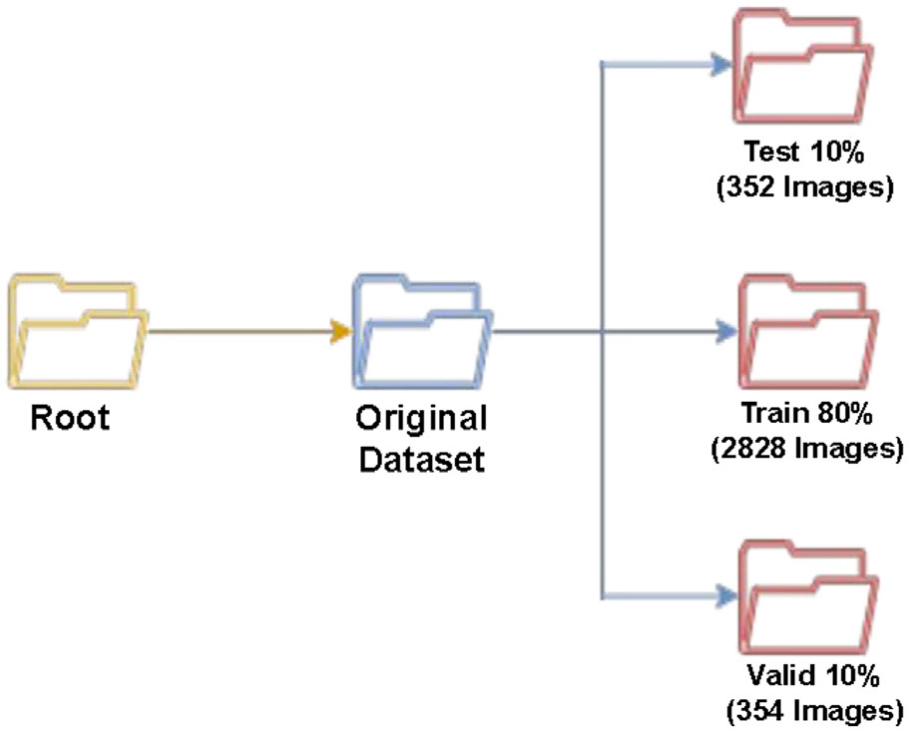
Dataset Folder Structure.

Furthermore, we have divided the dataset into train (80 %), test (10 %), and validation (10 %) subsets using Roboflow’s randomized split, which preserves class distribution across all subsets. This structured split allows the dataset to be readily used for training machine learning or deep learning models [8] without requiring any additional partitioning. In Fig. 5, we can visualize a simple structure for our dataset. Our dataset starts from the root folder, which is named the “Original Dataset”. All the image folders contain their Pascal VOC annotations with their respective images. From Fig. 5, we can observe that the original dataset contains a total of 3534 images. Afterwards, the original dataset is divided into 3 parts, namely train, test, and validation folders, which contain 2828, 352, and 354 images, respectively.

## 5 Experimental Design, Materials, and Methods

**Fig. 6:**
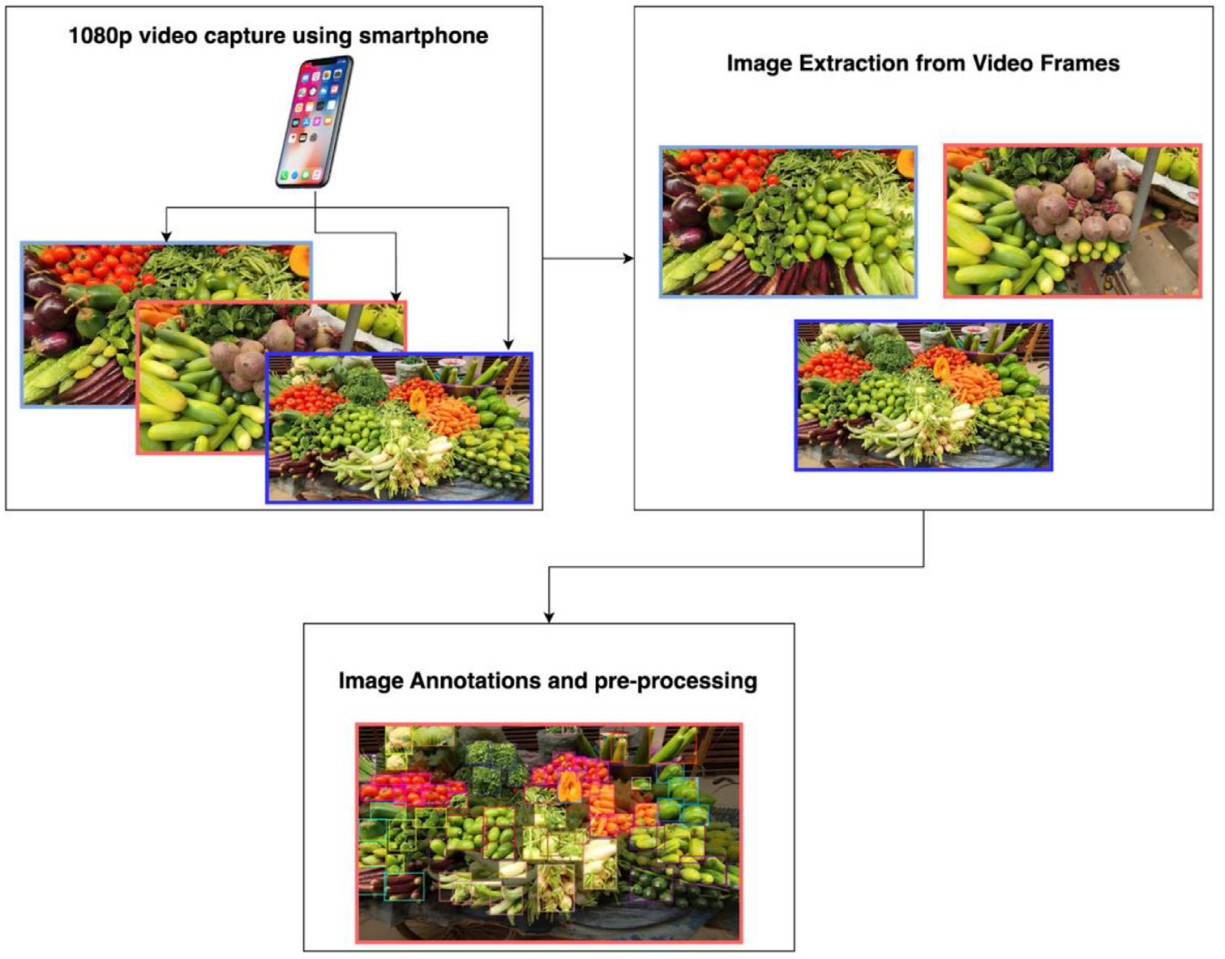
Schematic Representation of the Experimental Procedure.

The experimental setup for the image collection process involved certain important steps. Initially, the videos of the vegetables were captured [3] using a Redmi Note 12 Android smartphone equipped with a 50 MP triple camera system. The device in itself can capture high-resolution videos of 1080p and 30 fps (frames per second). These videos were taken from many street vegetable vendors. From the recorded videos, images were extracted in JPG format for better analysis. Afterwards, the extracted images were curated for annotation. The next part depicts the initial pre-processing of the images to standardize the resolution and ensure consistency across the dataset. The preprocessing was performed using the Roboflow platform. Fig. 6 offers a detailed depiction of the complete experimental setup process.

### Data collection pipeline

**Fig. 7:**
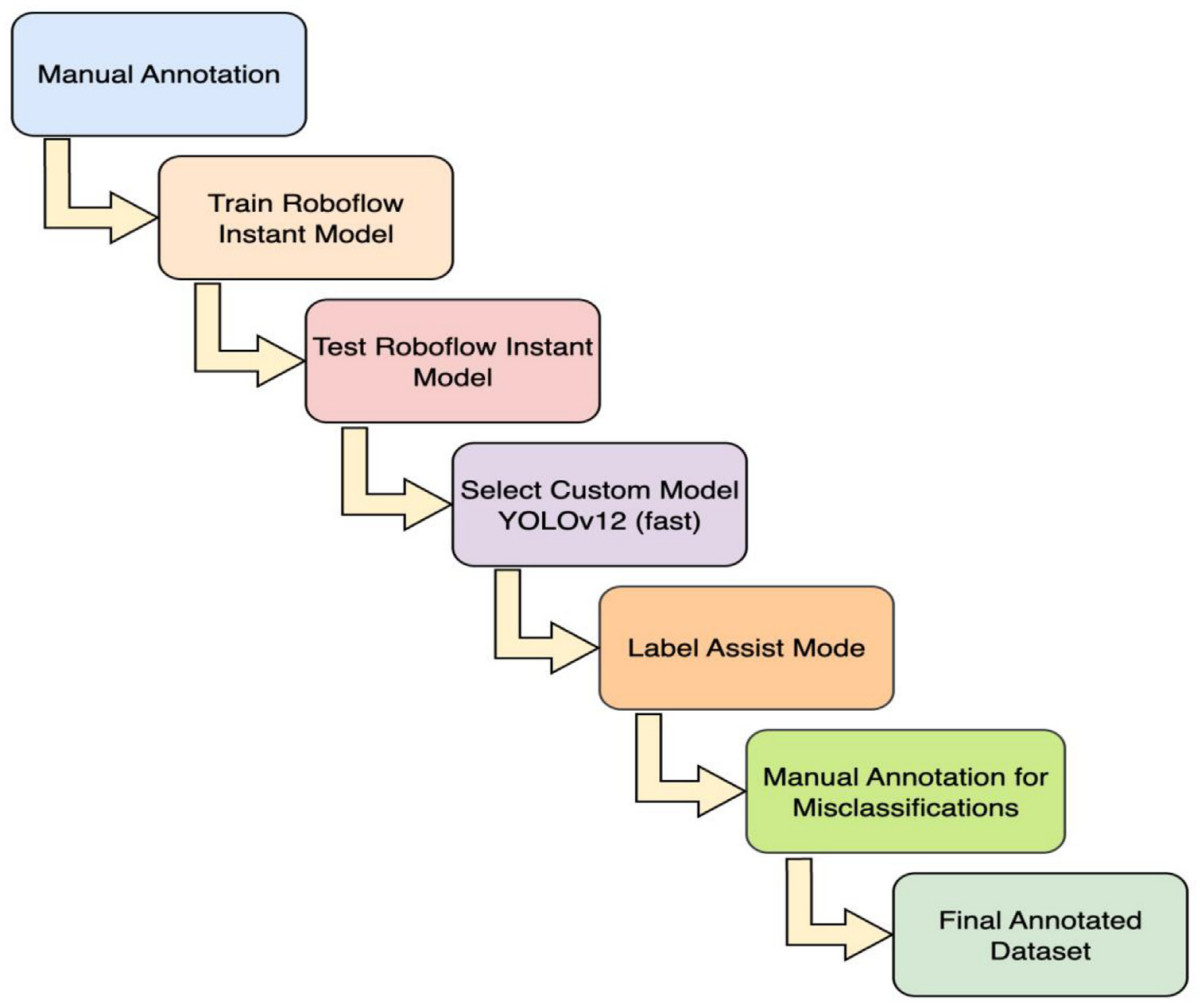
Workflow for dataset annotation and model-assisted labelling.

At first, we captured videos of vegetables from street vendor vehicles using a standard smartphone camera. The initial data collection starts from here and is divided into 2 stages: the image collection stage and the annotation stage. These 2 stages also have additional phases within them. Within the image collection stage, there are 2 phases where the videos of the vegetables were collected, and images were acquired from the videos. Moreover, the annotation stage also has two phases where the collected images are annotated using the Roboflow platform and then pre-processed techniques were used, finally preparing the total images for the final dataset. In the annotation pipeline, a subset of images was manually annotated to establish accurate ground truth labels. These annotations were initially used to train the Roboflow instant model. The Roboflow Instant model was tested to automatically label the remaining unannotated images; however, its performance on vegetable detection was unsatisfactory due to substantial misclassifications. To improve annotation quality, we selected the custom model training on the Roboflow platform, which was a YOLOv12 object detection (fast) model, and it was trained on the already labelled images. The model’s backbone was trained using default hyperparameters with default values including a batch size of 16, input image size of 640 × 640, initial learning rate of 0.01 with cosine scheduling, momentum of 0.937, weight deacy of 0.0005, dropout of 0.2, and standard YOLO-type augmentations such as RandAugment, random flipping (horizontal: 0.5), color jittering (hue: 0.015, saturation: 0.7, value: 0.4), random translation (0.1), erasing (0.4), and mosaic augmentation was enabled with a closing phase of 10 epochs. The trained model achieved a mAP@50 of 72.2 % with a precision of 69.7 % and a recall of 70.2 %, presenting optimal detection performance. Afterwards, the model was employed in Label Assist mode to generate annotations for the remaining unlabelled images. Predictions that were not generated by the model were subsequently manually labelled to ensure accurate annotation. Fig. 7 illustrates the overall annotation pipeline, where both manual and automated labelling approaches were integrated to generate the finalised dataset.

**Fig. 8:**
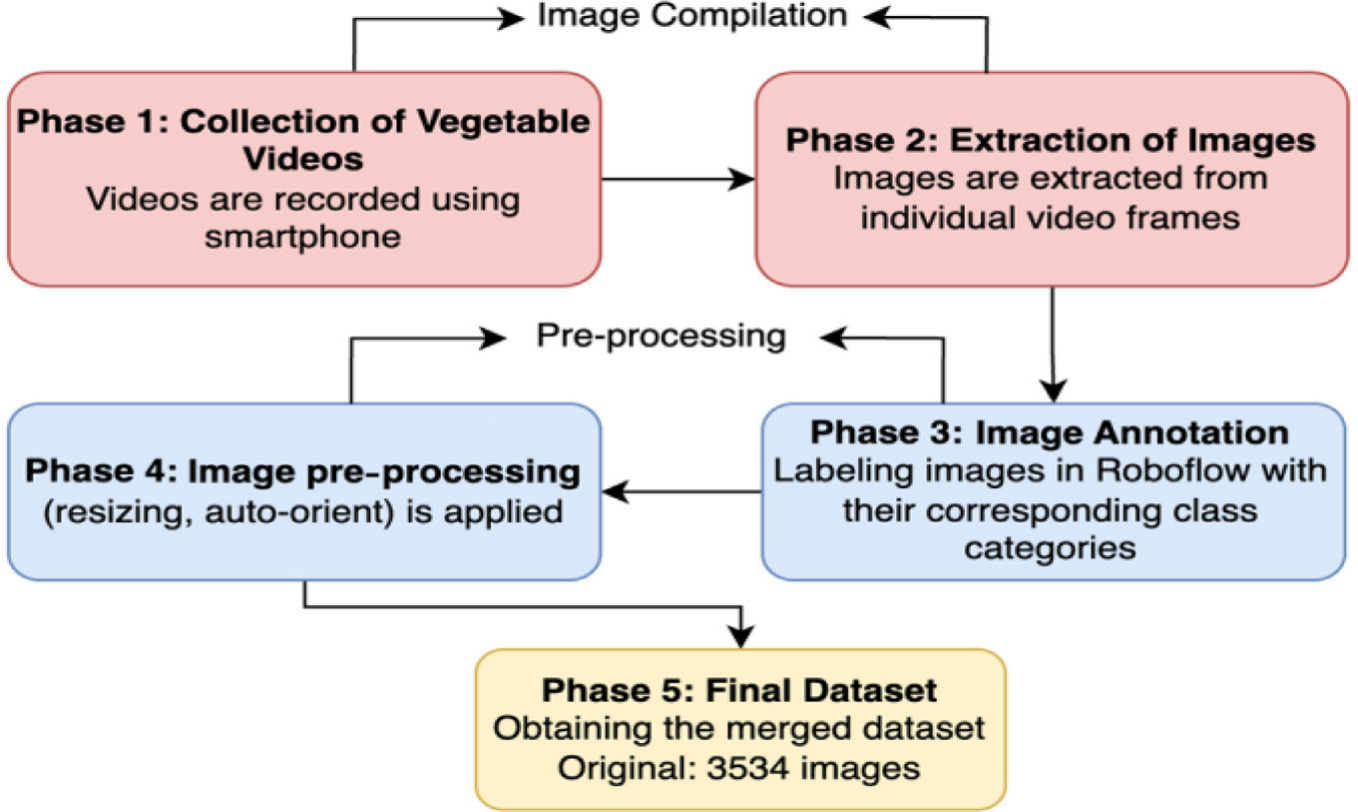
Two-stage Experimental Setup Outlining Five Key Phases.

In Fig. 8, we can observe the total process pipeline, where the whole pipeline consists of a total of 2 stages, containing 5 phases within them.

**Fig. 9:**
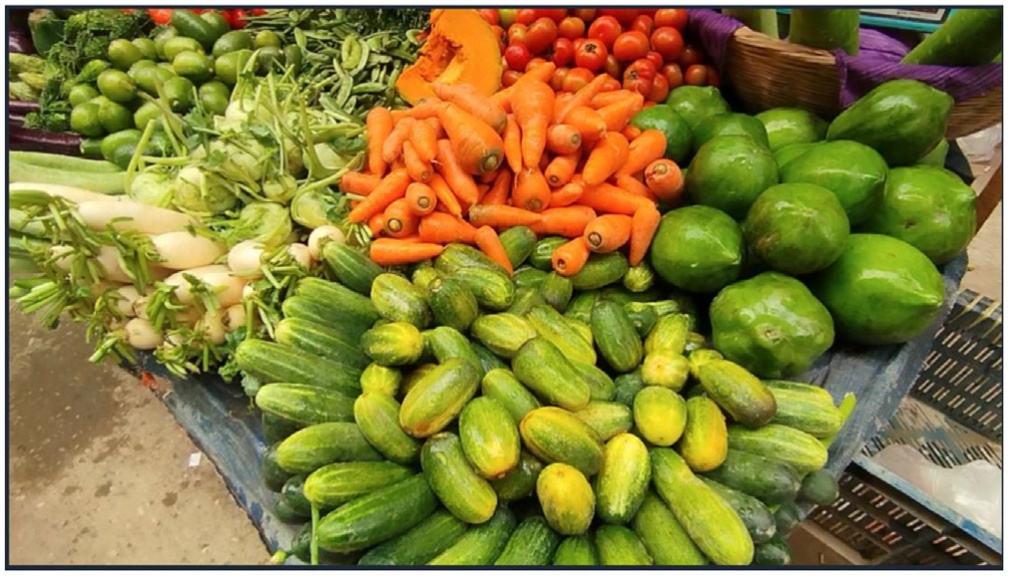
Extracted Image from the Video Footage.

For image acquisition, a standard smartphone camera was used. The videos of the vegetables captured on the phone were done at high resolution (1080p) at 30 frames per second. The primary data extracted from the footage was collected across multiple roadside vendor stalls in Banasree, Block# C, and images acquired from the videos were at different frames per second, depending on the images from the videos. A total of 9 videos, ranging from 1–4 min, were captured for our dataset. Fig. 9 illustrates a sample image that was extracted from the video footage.

### Image annotation

The next stage is the data annotation. The extracted images are annotated and refined to maintain correctness and minimize discrepancies in the dataset. The Roboflow platform was used to carefully review and adjust bounding box annotations, improving annotation quality and ensuring accurate labelling of the images.

### Image pre-processing

After all the images were extracted and annotated, we employed simple pre-processing techniques to make sure the dataset remains fully authentic in the final stage. The following pre-processing steps were involved:

### Resizing

The original images were captured at a resolution of 8160 × 6120 using the Redmi Note 12 and later resized to 640 × 640 pixels, which is a widely adopted standard resolution in object detection research. Resizing was performed after annotation to ensure reliability with bounding boxes. The “Fit within” method in the Roboflow platform was employed for resizing, which resizes each image to fit within the target dimensions while preserving the original aspect ratio, with padding added when required.

**Fig. 10:**
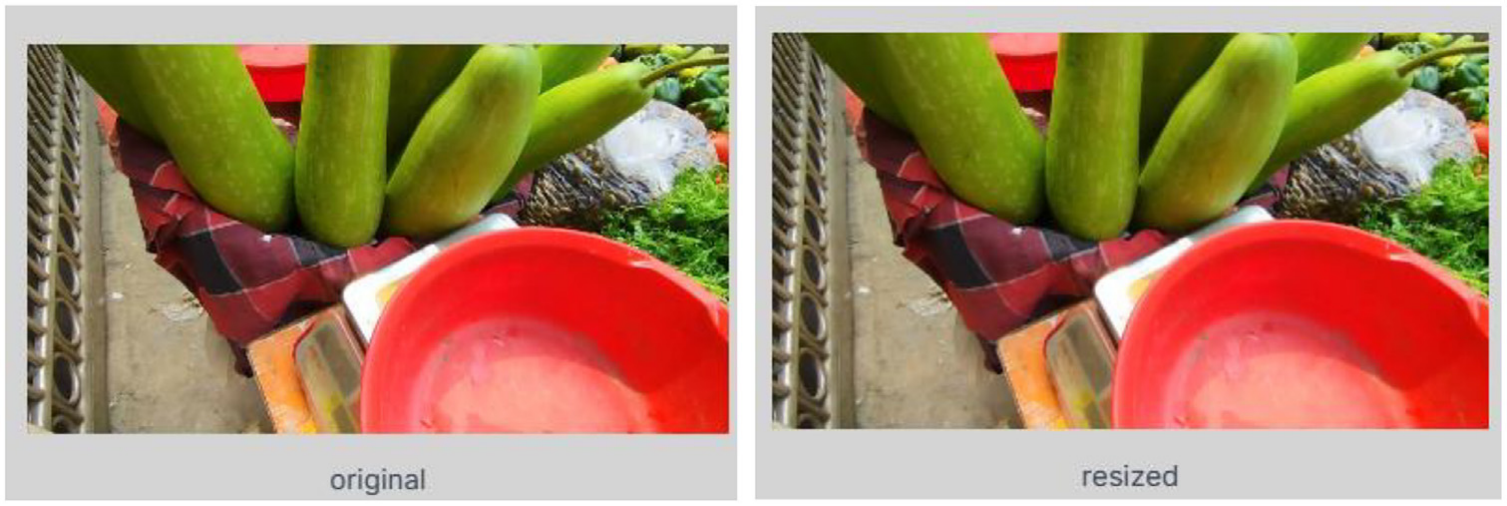
Visualization of Pre-processed Image from the Dataset.

Finally, after all the images were pre-processed, we organized the final dataset in a structured and ready-to-use format for model training. Fig. 10 shows the pre-processed images in the dataset.

## Limitations

Alongside rich annotations and standard illumination conditions, the data set also has some limitations that might, at the very least, impact its usability and generalizability:

• **Geographic Coverage:** Data were gathered for street vendors in one urban area in Dhaka, Bangladesh, and may limit the generalizability of trained models to other agricultural areas with different climates, production patterns, or supply chains. Data collection across multiple sites would introduce geographic diversity.
• **Size of dataset:** The dataset is modest with few images (3534), which would likely be insufficient to train very sophisticated deep models without transfer learning or data augmentation.
• **Device Dependence:** All the images were taken with one specific model of smartphone camera. Image quality and feature differences from other cameras or devices can affect the robustness of a model.
• **Temporal and Seasonal Coverage:** The dataset does not include images that cover temporal differences, such as different seasons or growth stages of the crops, which may reduce model effectiveness over time or at different growth stages.
• **Annotation Scope:** Annotations focus on vegetable identification and localization but do not include labels for diseases, pests, or other crop health indicators, limiting direct use in crop health monitoring without additional labelling.
• **Environmental Conditions:** Although images were taken under broad daylight, certain extreme weather or environmental conditions (e.g., heavy rain, fog, night-time) are not represented, potentially affecting model performance under those conditions.

## Ethics Statement

The authors adhere to the journal's ethical guidelines and confirm that this research does not involve humans, animals, or data obtained from social media. The datasets utilized in the study are publicly accessible, and appropriate citation protocols should be followed when utilizing these datasets.

## Credit Author Statement

**Sabrina Jahan**:Conceptualization, Methodology, Supervision, Visualization, Project administration, Validation, Writing – review & editing; **B. M. Shahria Alam**: Investigation, Data collection, Methodology, Writing – original draft, Writing – review & editing; **Ishraque Manzur**: Conceptualization, Methodology, Supervision, Visualization, Project administration, Validation, Writing – review & editing; **Tawhidur Rahman**: Conceptualization, Methodology, Supervision, Visualization, Project administration, Validation, Writing – review & editing; **Mahamudul Hasan**: Investigation, Data collection, Methodology, Writing – original draft, Writing – review & editing; **Raiyan Gani**: Investigation, Data collection, Writing – original draft, Writing – review & editing; **Md Miskat Hossain**: Investigation, Data collection, Methodology, Writing – original draft, Writing – review & editing; **Mohammad Rifat Ahmmad Rashid**: Conceptualization, Methodology, Supervision, Visualization, Project administration, Validation, Writing – review & editing.

## Data Availability

Mendeley DataVegetable Object Detection Dataset from Bangladesh (Original data)
